# Prognostic utility of the MECKI score in a mixed United States cohort

**DOI:** 10.14814/phy2.70770

**Published:** 2026-02-11

**Authors:** Adarsh Mallepally, Kaivalya Dandamudi, Matthew G. Kaye, Teymur Zavar, Bailey Parsons, Shreya Krishnamurthy, Hamang Patel, Ross Arena, Justin M. Canada, Cory R. Trankle

**Affiliations:** ^1^ School of Medicine Virginia Commonwealth University Richmond Virginia USA; ^2^ Department of Internal Medicine Virginia Commonwealth University Richmond Virginia USA; ^3^ Division of Cardiology, VCU Pauley Heart Center Virginia Commonwealth University Richmond Virginia USA; ^4^ College of William & Mary Williamsburg Virginia USA; ^5^ Virginia Commonwealth University Richmond Virginia USA; ^6^ Department of Physical Therapy, College of Applied Health Sciences University of Illinois Chicago Chicago Illinois USA

**Keywords:** cardiopulmonary exercise test, heart failure, prognosis

## Abstract

The Metabolic Exercise test data combined with Cardiac and Kidney Indexes (MECKI) score has demonstrated prognostic utility in European and Asian cohorts with heart failure with reduced ejection fraction (HFrEF). We sought to evaluate its performance in an American cohort. We retrospectively identified patients who underwent cardiopulmonary exercise testing (CPX) at our institution in 2022–2024 with data to calculate the MECKI and CPX Risk scores. The primary endpoint was a composite of death, heart failure admission, heart transplantation, or ventricular assist device. Survival analysis was assessed via Kaplan–Meier curves and log‐rank test, with ROC curves for comparison. Overall, 803 patients met criteria, with 451 (56%) female, 228 (28%) Black race, and median body mass index 29.4 (25.0–34.2) kg/m^2^. Pre‐existing HFrEF was present in 187 (23%) patients. 719 (90%), 41 (5%), and 43 (5%) patients achieved MECKI scores <10%, 10%–20%, and ≥20%, respectively, with stepwise increases in 2‐year risk of primary endpoints (log‐rank χ^2^ = 196.0, *p* < 0.001). ROC curves demonstrated better performance of MECKI scores compared to CPX Risk scores. Events were similarly predicted in patients with HFrEF, with similar performances between the two scores. In conclusion, in a mixed American cohort the MECKI score demonstrated robust performance in predicting event‐free survival.

## 1 INTRODUCTION

Heart failure (HF) is a multifactorial clinical syndrome that imposes a significant burden on healthcare in the United States (U.S.), detrimentally impacting quality of life, increasing hospitalization rates, and driving cost of care higher (Salah et al., 2022; Savarese et al., 2023). The prevalence of HF is projected to surge to 10.3 million Americans by 2040 based on the aging population, making it an increasingly important public health issue (Bozkurt et al., 2025). Despite recent advances in HF therapies, mortality rates have continued to rise, effectively reversing the progress made during the 2000s.

Additionally, upwards of 500,000 Americans may benefit from heart transplant or left ventricular assist device (LVAD) implantation, but shortages in organ donor supply and subjective interpretation of criteria can complicate candidate selection for such advanced HF management strategies (Miller et al., 2019; Samman‐Tahhan et al., 2018). Hence, accurate risk stratification would be immensely beneficial for allocating resources more effectively and reducing waitlist mortality. Altogether, these trends underscore a critical gap in reliable prognostic estimates, particularly for chronic HF, which likely stems from the unpredictable variance in disease course between patients and the complex interplay of comorbidities (Jones et al., 2019).

Several multivariable prognostic risk scores have been developed in the past few decades to address this unmet need for HF patients (Jia et al., 2024; Savarese et al., 2022). One such tool, the Metabolic Exercise test data combined with Cardiac and Kidney Indexes (MECKI) score, was developed by Agostoni et al. (2013) to identify risk of cardiovascular death and urgent heart transplant using patients recruited from established HF centers in Italy. The MECKI score incorporates ventilatory expired gas parameters derived from cardiopulmonary exercise testing (CPX) as well as echocardiographic data and markers of comorbidities and fluid imbalance. Integrating these independent predictors of prognosis offered the strongest discrimination ability and proved to outperform other commonly used models, including the Heart Failure Survival Score (HFSS), Seattle Heart Failure Model (SHFM), and Meta‐analysis Global Group in Chronic Heart Failure (MAGGIC) (Adamopoulos et al., 2023; Agostoni et al., 2013; Freitas et al., 2017). The MECKI score was most recently validated by Adamopoulos et al. (2023) in patients with HF with reduced ejection fraction (HFrEF) within a European and Asian cohort, reaffirming its prognostic capabilities among high‐risk populations. Despite the MECKI score offering a common ground to compare patients between institutions, there is a dearth of MECKI validation studies performed in a diverse U.S. cohort, limiting its implementation in clinical practice within the country (Salvioni et al., 2020).

To this end, we sought to evaluate the performance of the MECKI score in a U.S. cohort of patients both with and without HFrEF. We also aimed to compare the prognostic capabilities of the MECKI score to another established multivariate HF model, the CPX Risk score, an aggregate that relies solely on CPX parameters and has previously been validated among a multicenter international cohort (Myers et al., 2008).

## 2 MATERIALS AND METHODS

### 2.1 Study design and population

We retrospectively identified adult patients who underwent first CPX at the Virginia Commonwealth University Health System from 2022 to 2024 who had sufficient data to calculate MECKI and CPX Risk scores. Demographics, medical history, prescribed medications, laboratory findings, CPX data, echocardiographic data, and downstream clinical outcomes were collected from electronic medical records. The study was approved by the Virginia Commonwealth University institutional review board, with a waiver of consent due to the retrospective nature of the study. The study was performed in accordance with the Declaration of Helsinki.

### 2.2 Clinical data

We recorded any prior diagnosis of HF, history of congenital heart disease, and other relevant medical comorbidities listed in the medical records and confirmed by clinical exercise physiologist interview on the day of CPX. Laboratory findings available in the electronic record were recorded, including hemoglobin, serum sodium, and serum creatinine levels. Left ventricular ejection fraction (LVEF) was recorded from the most recent clinical report from transthoracic echocardiography prior to CPX. Patients with a prior documented history of HF with LVEF <40% and/or with the most recent echocardiogram measuring LVEF ≤45% were categorized as HFrEF for dedicated subgroup analysis.

### 2.3 Cardiopulmonary exercise testing

All patients performed a symptom‐limited, maximal effort, CPX using a treadmill or upright bicycle ergometer interfaced with a metabolic cart for clinical evaluation. Exercise protocols were chosen at the discretion of the clinical exercise physiologist as per institutional protocols in part based on the Duke Activity Status Index response to target an exercise duration of 8–12 min (Canada et al., 2021). In addition to standard hemodynamic data, routinely reported measurements during CPX at our institution include: peak oxygen consumption (VO_2_, defined as the highest 10‐s rolling average in the last 30 s of exercise) expressed as an absolute value (mL•min^−1^) and relative to body weight (mL •kg^−1^•min^−1^), ventilatory efficiency as represented by the minute ventilation to carbon dioxide production (VE/VCO_2_) slope to peak exercise, heart rate recovery (defined as maximal heart rate minus heart rate at 1 min in recovery), resting end‐tidal CO_2_ pressure (derived from the average of a 2‐min sitting resting period before the test), and oxygen uptake efficiency slope (calculated using VO_2_ = m * [log_10_VE] + b, where m = the oxygen uptake efficiency slope). A CPX Risk score was obtained by summing the specific weightings established by Myers et al. (2013) that are assigned to each of the above CPX variables. As such, a VE/VCO_2_ slope ≥ 34, heart rate reserve ≤ 6 beats, oxygen uptake efficiency slope ≤ 1.4, partial pressure of end tidal CO_2_ < 33 mmHg, and peak VO_2_ ≤ 14.4 mL •kg^−1^•min^−1^ were assigned weightings of 7, 5, 3, 3, and 2, respectively (Table S1).

### 2.4 MECKI score

The percent‐predicted peak VO_2_ and estimated glomerular filtration rate using the Modification of Diet in Renal Disease equation, without including self‐identified race, were calculated in accordance with the original validation study. To ensure an accurate comparison between exercise modalities, the peak VO_2_ data measured on the treadmill was reduced by 10% (Adamopoulos et al., 2023; Agostoni et al., 2013; Salvioni et al., 2020). Taking these measurements together, a MECKI score expressed as a percentage was generated. All MECKI calculations performed are listed in Table S1.

### 2.5 Patient follow‐up

Following CPX, subsequent clinical events were recorded from the electronic medical record. The primary endpoint was a composite of death, heart failure hospitalization (defined as admission to the hospital for a primary diagnosis of decompensated heart failure requiring the use of intravenous diuretics), heart transplantation, or left ventricular assist device implantation.

### 2.6 Statistical analysis

Categorical variables are represented as *n* (%). The Kolmogorov–Smirnov test was used to assess for normality of continuous variables, finding significant departure from normal distributions in most of the data. As such, continuous data is expressed as median (interquartile range). Comparisons among MECKI score subgroups were performed using chi‐square test and independent samples Kruskal–Wallis test for categorical variables and continuous variables, respectively. Survival analysis between subgroups was assessed via Kaplan–Meier curves and log‐rank test to determine differences in median event‐free survival, stratifying the cohort according to previously reported cutoff values for each (i.e., MECKI scores of 10% and 20%, CPX scores of 5, 10, and 15). Findings were considered significant if *p* < 0.05. Receiver operating characteristic (ROC) curves and area under the ROC curve (AUC) analyses were utilized to evaluate the ability of MECKI and CPX Risk scores to predict incident events through 2 years of follow‐up. From these curves, Youden's index was used to identify optimal cutoff values within our cohort for each score, repeating the Kaplan–Meier curves and calculating test performance. Analyses were performed using SPSS version 29.0 (IBM, Armonk, NY).

## 3 RESULTS

### 3.1 Baseline characteristics

In total, 1243 adult patients underwent CPX at our institution from 2022 to 2024, of whom 803 (65%) had sufficient data available to calculate both MECKI and CPX summed risk scores and thus were included in the study cohort. Clinical characteristics including patient demographics, medical history, laboratory values, echocardiographic data, CPX data, and MECKI scores are reported in Table 1. Overall, the cohort included 451 (56%) females and 228 (28%) individuals of self‐identified Black race. Median age at the time of CPX was 52 (37–66) years, with a median body mass index of 29.4 (25.0–34.2) kg/m^2^. There were 332 (41%) patients with a pre‐existing diagnosis of HF, including 187 (23% of the overall cohort) with a history of HF with ejection fraction ≤45%. Additionally, 197 (25%) patients had a history of congenital heart disease.

**TABLE 1:** Clinical characteristics and CPX performances of entire cohort.

	Total study cohort	MECKI <10%	MECKI 10%–20%	MECKI ≥20%	*p* Value
	(*n* = 803)	(*n* = 719)	(*n* = 41)	(*n* = 43)	
Demographics					
Age at CPX (years)	52 (37–66)	51 (35–66)	59 (50–65)	58 (46–66)	0.070
Female sex	451 (56%)	430 (60%)	13 (32%)	8 (19%)	<0.001
Race					
White	519 (65%)	486 (68%)	22 (54%)	11 (26%)	<0.001
Black	228 (28%)	182 (25%)	15 (37%)	31 (72%)	
Other/not listed	1 (2%)	51 (7%)	4 (10%)	1 (2%)	
Body mass index (kg/m^2^)	29.4 (25.0–34.2)	29.6 (25.1–34.5)	27.2 (24.2–31.7)	25.7 (22.8–32.7)	0.010
Medical history					
Heart failure diagnosis	332 (41%)	253 (35%)	38 (93%)	41 (95%)	<0.001
HFrEF ≤45%	187 (23%)	111 (15%)	35 (85%)	41 (95%)	<0.001
Left ventricular ejection fraction (%)	57.5 (47.5–62.5)	57.5 (52.5–62.5)	27.5 (19.0–36.3)	22.5 (17.5–27.5)	<0.001
Congenital heart disease	197 (25%)	195 (31%)	2 (7%)	0	<0.001
Coronary artery disease	212 (27%)	170 (24%)	19 (46%)	23 (54%)	<0.001
Hypertension	457 (57%)	384 (54%)	33 (81%)	40 (93%)	<0.001
Hyperlipidemia	392 (49%)	336 (47%)	30 (73%)	26 (61%)	0.001
Diabetes mellitus	187 (23%)	149 (21%)	20 (49%)	18 (42%)	<0.001
Atrial fibrillation or flutter	172 (22%)	150 (21%)	8 (20%)	14 (33%)	0.188
Chronic kidney disease	168 (21%)	114 (16%)	20 (49%)	34 (79%)	<0.001
Chronic obstructive pulmonary disease	61 (8%)	49 (7%)	4 (10%)	8 (19%)	0.016
History of smoking	243 (30%)	200 (28%)	18 (44%)	25 (58%)	<0.001
Cardiac implantable electronic device	176 (22%)	131 (18%)	21 (51%)	24 (56%)	<0.001
Medications					
Beta blocker	378 (47%)	317 (44%)	24 (59%)	37 (86%)	<0.001
RAAS inhibitor	338 (42%)	283 (39%)	27 (66%)	28 (65%)	<0.001
Mineralocorticoid antagonist	175 (22%)	139 (19%)	18 (44%)	18 (42%)	<0.001
Nondihydropyridine calcium channel blocker	22 (3%)	22 (3%)	0	0	0.267
Dihydropyridine calcium channel blocker	115 (14%)	101 (14%)	5 (12%)	9 (21%)	0.422
Loop diuretic	228 (28%)	174 (24%)	24 (59%)	30 (70%)	<0.001
SGLT2 inhibitor	143 (18%)	105 (15%)	21 (51%)	17 (40%)	<0.001
Laboratory values					
Hemoglobin (g/dL)	13.4 (12.1–14.6)	13.5 (12.3–14.6)	12.5 (10.9–14.3)	12.4 (10.7–14.4)	0.003
Sodium (mmol/L)	139 (138–140)	139 (138–140)	138 (137–140)	138 (135–139)	<0.001
Creatinine (mg/dL)	0.9 (0.8–1.2)	0.9 (0.7–1.1)	1.2 (1.0–1.9)	2.0 (1.4–6.3)	<0.001
Estimated glomerular filtration rate	71 (50–98)	82.3 (65.1–101.3)	62.5 (30.6–75.5)	34.2 (9.5–57.3)	<0.001
CPX and MECKI data					
Peak VO_2_ peak (mL •kg^−1^•min^−1^)	17.0 (12.5–22.1)	17.7 (13.0–22.9)	13.3 (10.9–16.9)	12.0 (8.2–14.2)	<0.001
Percent predicted peak VO_2_ (%)	71 (56–89)	75 (61–92)	51 (40–58)	38 (29–50)	<0.001
VE/VCO_2_ slope	30.9 (27.3–35.6)	30.4 (27.0–34.2)	36.0 (33.1–40.9)	40.2 (35.0–48.1)	<0.001
CPX Risk score	5 (3–10)	5 (3–10)	12 (7–17)	15 (10–20)	<0.001
MECKI score (%)	0.6 (0.3–2.6)	0.5 (0.2–1.4)	13.6 (11.2–16.2)	32.7 (24.5–47.4)	<0.001
CPX referral reason					
HF prognosis	142 (18%)	95 (13%)	24 (59%)	23 (54%)	<0.001
Dyspnea	236 (29%)	230 (32%)	4 (10%)	2 (5%)	
Other	425 (53%)	394 (55%)	18 (42%)	18 (42%)	

*Note*: Data is presented as median (interquartile range) or number (%). Values are medians (interquartile range) or number (%). Comparisons made across the three subgroups using chi‐square test or independent samples Kruskal–Wallis test.

Abbreviations: CPX, cardiopulmonary exercise test; RAAS, renin‐angiotensin‐aldosterone system; SGLT2, sodium‐glucose cotransporter 2; VE/VCO_2_, minute ventilation to carbon dioxide production; VO_2_, oxygen consumption.

### 3.2 CPX performances and risk scores

In the total cohort, median peak VO_2_ was 17.0 (12.5–22.1) mL •kg^−1^•min^−1^, representing 71% (56%–89%) of predicted values (Table 1). Median MECKI score was 0.6% (0.3%–2.6%), including 719 (90%), 41 (5%), and 43 (5%) patients who achieved MECKI scores <10%, 10%–20%, and ≥20%, respectively. Male sex, self‐identified Black race, and the presence of several comorbidities—particularly HF diagnoses—were more common across escalating MECKI score subgroups, and progressively worse CPX‐derived measurements were similarly noted. Median CPX Risk scores were 5 (3–10), again with stepwise increases across MECKI subgroups.

### 3.3 Survival analysis

**FIGURE 1:**
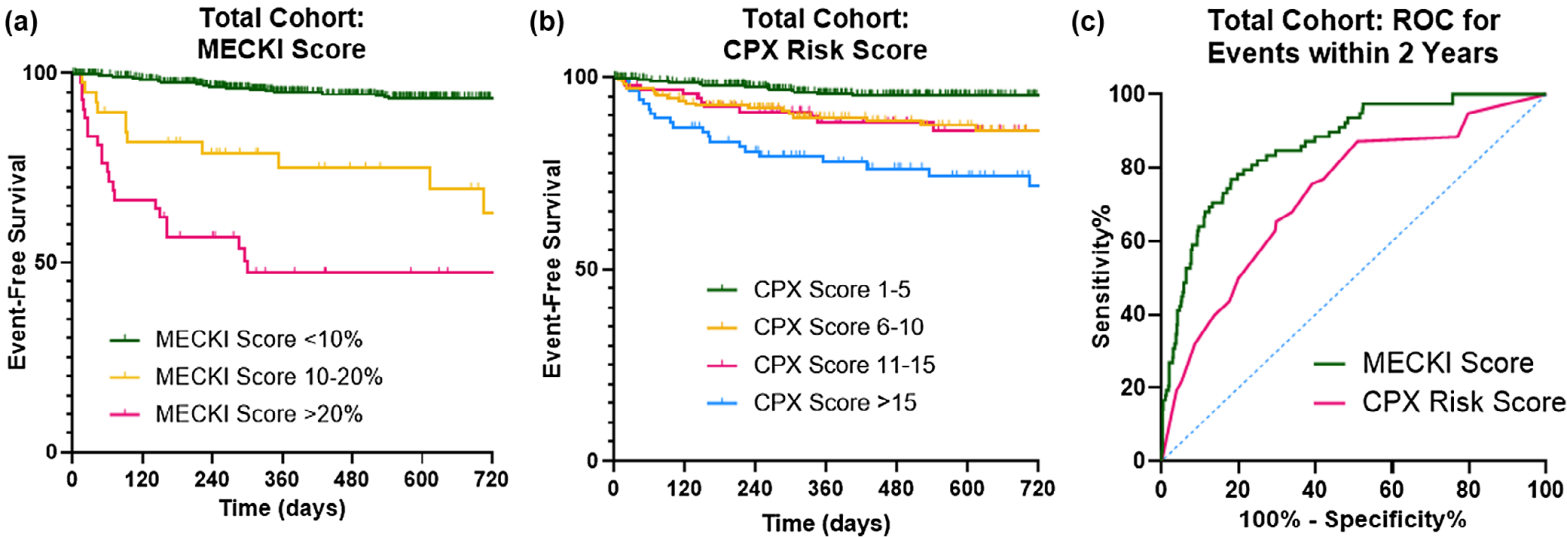
Kaplan–Meier event‐free survival curves (using established cutoff values) and ROC curves in the total cohort. Both the MECKI score (a) and CPX Risk score (b) allowed for discrimination of risk among the entire cohort using previously established cutoff values. On receiver operating characteristic curve analysis (c), the MECKI score had superior performance.

Median follow up duration was 513 (261–843) days, over which time there were 86 patients who experienced the primary endpoint, including 29 patients who expired, 64 who were admitted with acutely decompensated HF, 11 who underwent heart transplantation, and four who received left ventricular assist devices. When stratifying the entire cohort according to previously established MECKI score cutoffs, patients had stepwise decreases in event‐free survival (log‐rank χ2 = 196.0, *p* < 0.001, Figure 1). Similarly, stratifying the cohort according to previously established CPX Risk score cutoffs discriminated among risk of lower event‐free survival (log‐rank χ2 = 47.1, *p* < 0.001, Figure 1).

Receiver operating curves are also shown in Figure 1, demonstrating a higher performance of MECKI scores compared to CPX Risk scores for predicting 2‐year outcomes in the total cohort (AUC = 0.861 [95% CI 0.820–0.903] vs. AUC = 0.728 [95% CI 0.669–0.788]). Using Youden's index, optimal cutoff values of MECKI >2.78 and CPX score >6 were identified. Survival curves using these new cutoff values are shown in Figure S1, with test performances for accurately predicting adverse events within 2 years shown in Table S2. The sensitivity and specificity of MECKI scores were 77% and 82%, respectively, yielding an accuracy of 81% (compared to sensitivity of 76%, specificity of 62%, and accuracy of 62% for the CPX Risk score).

### 3.4 Subgroup analysis in patients with systolic heart failure

**FIGURE 2:**
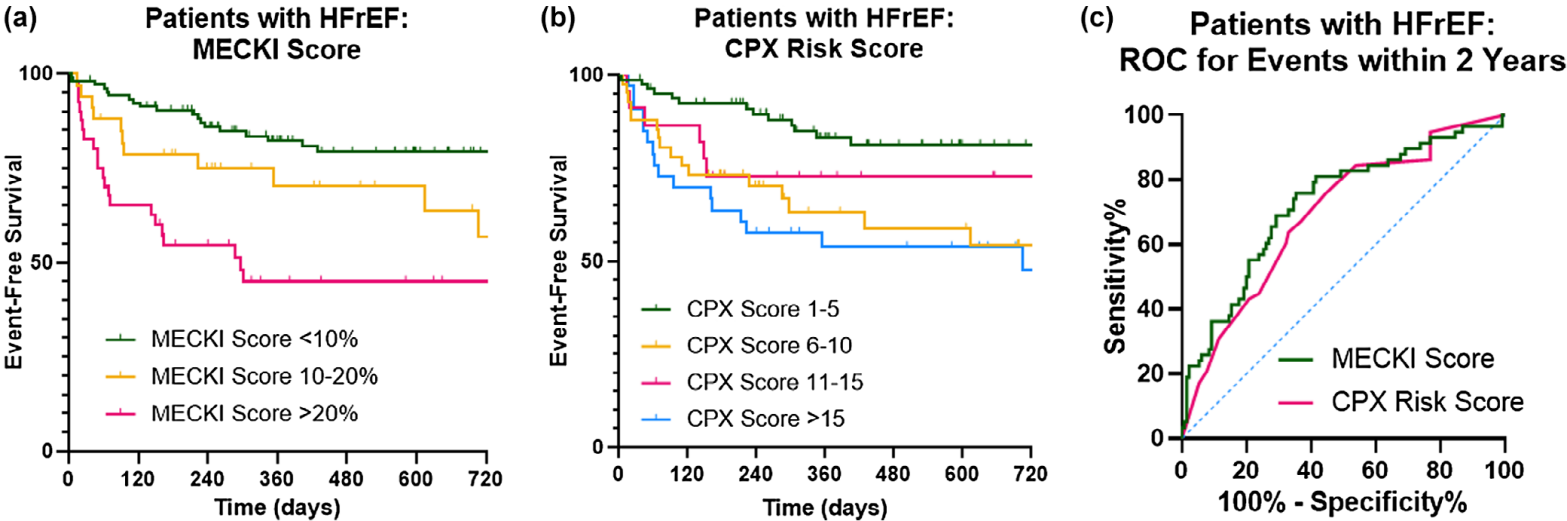
Kaplan–Meier event‐free survival curves (using established cutoff values) and ROC curves in patients with HFrEF. The MECKI score (a) and CPX Risk score (b) also discriminated among the subgroup of patients with heart failure with reduced ejection fraction. Receiver operating characteristic curves (c) were similar between the two risk scores.

Clinical characteristics for the subgroup of patients with HFrEF (*n* = 187, or 18% of the total study cohort) are shown in Table 2, stratified according to MECKI score cutoffs, with performance metrics from CPX and risk scores also reported. Using the same previously established cutoff values, the MECKI score again showed good discriminatory power (log‐rank χ2 = 26.6, *p* < 0.001, Figure 2). Similarly, the previously established CPX Risk score cutoffs allowed for discrimination of downstream composite events (log‐rank χ2 = 14.0, *p* = 0.003). Receiver operating curves for 2‐year composite events are displayed in Figure 2, with similar performances in this subgroup between the two risk scores: AUC 0.725 (95% CI 0.645–0.806, *p* < 0.001) for the MECKI score versus AUC 0.692 (95% CI 0.611–0.773, *p* < 0.001). Using Youden's index, optimal cutoff values of MECKI score ≥7.80 and CPX Risk score >5 were established. Survival curves and test performances with these new cutoff values are displayed in Figure S2 and Table S3. These cutoff values yielded a sensitivity of 76%, specificity of 65%, and overall accuracy of 68% for the MECKI score (compared to 76%, 55%, and 62%, respectively, for the summed CPX Risk score).

**TABLE 2:** Clinical characteristics and CPX performances of patients with systolic heart failure.

	All patients with HFrEF	MECKI <10%	MECKI 10%–20%	MECKI ≥20%	*p* Value
	(*n* = 188)	(*n* = 112)	(*n* = 35)	(*n* = 41)	
Demographics					
Age at CPX (years)	56 (45–64)	53 (44–61)	60 (49–65)	58 (45–66)	0.160
Female sex	65 (35%)	47 (42%)	10 (29%)	8 (20%)	0.025
Race					
White	91 (48%)	63 (56%)	17 (49%)	11 (27%)	0.004
Black	85 (45%)	42 (38%)	14 (40%)	29 (71%)	
Other/not listed	12 (6%)	7 (6%)	4 (11%)	1 (2%)	
Body mass index (kg/m^2^)	30.6 (25.7–34.3)	31.5 (28.1–35.1)	27.2 (24.0–31.4)	26.9 (23.2–32.9)	<0.001
Medical history					
Left ventricular ejection fraction (%)	27.5 (22.5–37.5)	32.5 (25.6–39.7)	22.5 (17.5–32.5)	22.5 (17.5–27.3)	<0.001
Congenital heart disease	11 (7%)	9 (10%)	2 (8%)	0	0.130
Coronary artery disease	83 (44%)	43 (38%)	17 (49%)	23 (56%)	0.125
Hypertension	140 (75%)	74 (66%)	28 (80%)	38 (93%)	0.003
Hyperlipidemia	116 (62%)	64 (57%)	28 (80%)	24 (59%)	0.047
Diabetes mellitus	73 (39%)	38 (34%)	17 (49%)	18 (44%)	0.226
Atrial fibrillation or flutter	56 (29%)	35 (31%)	7 (20%)	14 (34%)	0.352
Chronic kidney disease	79 (42%)	30 (27%)	16 (46%)	33 (81%)	<0.001
Deep vein thrombosis	14 (7%)	8 (7%)	2 (6%)	4 (10%)	0.785
Chronic obstructive pulmonary disease	17 (9%)	8 (7%)	2 (6%)	7 (17%)	0.124
History of smoking	78 (42%)	41 (37%)	14 (40%)	23 (56%)	0.094
Cardiac implantable electronic device	113 (60%)	69 (62%)	20 (57%)	24 (59%)	0.871
Medications					
Beta blocker	155 (82%)	98 (88%)	21 (60%)	36 (88%)	<0.001
RAAS inhibitor	150 (78%)	95 (85%)	27 (77%)	28 (68%)	0.072
Mineralocorticoid antagonist	111 (59%)	75 (67%)	18 (51%)	18 (44%)	0.022
Nondihydropyridine calcium channel blocker	0	0	0	0	N/A
Dihydropyridine calcium channel blocker	15 (8%)	6 (5%)	2 (6%)	7 (17%)	0.052
Loop diuretic	110 (59%)	59 (53%)	21 (60%)	30 (73%)	0.073
SGLT2 inhibitor	106 (56%)	68 (61%)	21 (60%)	17 (42%)	0.093
Laboratory values					
Hemoglobin (g/dL)	13.6 (11.8–14.8)	14.1 (12.6–15.3)	13.4 (11.0–14.3)	12.4 (10.8–14.3)	<0.001
Sodium (mmol/L)	139 (137–140)	139 (137–140)	138 (137–140)	138 (135–139)	0.003
Creatinine (mg/dL)	1.2 (0.9–1.6)	1.0 (0.8–1.3)	1.2 (1.0–1.6)	2.0 (1.4–6.5)	<0.001
Estimated glomerular filtration rate	65.7 (38.7–82.8)	73.9 (55.7–92.0)	64.7 (35.1–77.5)	34.2 (8.7–57.0)	<0.001
CPX and MECKI data					
Peak VO_2_ (mL •kg^−1^•min^−1^)	15.4 (12.1–19.3)	17.7 (13.6–21.4)	14.7 (12.2–17.3)	12.0 (8.8–14.4)	<0.001
Percent predicted VO_2_ (%)	61 (48–75)	71 (61–84)	62 (45–61)	40 (30–50)	<0.001
VE/VCO_2_ slope	32 (28–38)	29.3 (26.6–32.9)	35.4 (31.5–39.4)	40.2 (34.9–47.0)	<0.001
CPX Risk score	7 (3–13)	3 (0–8)	10 (6–17)	15 (10–20)	<0.001
MECKI score (%)	7.0 (2.8–17.1)	3.3 (1.7–6.1)	14.1 (11.6–16.5)	32.9 (24.4–47.7)	N/A
CPX referral reason					
HF prognosis	120 (64%)	73 (65%)	24 (67%)	23 (56%)	0.605
Dyspnea	8 (4%)	5 (5%)	2 (6%)	1 (2%)	
Other	60 (32%)	34 (30%)	9 (26%)	17 (42%)	

*Note*: Data is presented as median (interquartile range) or number (%). Values are medians (interquartile range) or number (%). Comparisons made across the three subgroups using chi‐square test or independent samples Kruskal–Wallis test.

Abbreviations: CPX, cardiopulmonary exercise test; RAAS, renin‐angiotensin‐aldosterone system; SGLT2, sodium‐glucose cotransporter 2.

## 4 DISCUSSION

In this retrospective analysis of an American cohort undergoing first‐time CPX, we had three key findings. For one, the MECKI score performed well in the identification of individuals at high risk for downstream adverse outcomes in the total cohort, encompassing patients with and without HFrEF. Secondly, its performance was superior to that of the commonly used CPX summed risk score. Third, in the subgroup of patients with HFrEF, the performance of the MECKI score was again high, albeit with limited improvement over the CPX summed risk score on ROC analysis.

To our knowledge, this is the first study to evaluate the performance of the MECKI score in predicting adverse HF related outcomes using patients from the U.S. The MECKI score was originally developed using data collected from an Italian population sample of patients with HFrEF, and its validation was later expanded to include a broader European and Asian HFrEF cohort. Until now, however, the prognostic implications of the MECKI score had not been validated in a broad U.S.‐based population.

Our findings have potentially significant clinical implications, as the population sampled herein includes patients both with and without HF as well as from different racial and ethnic backgrounds from prior investigations, including individuals who identified as Black (28%). Our population sample also included a higher proportion of females (56%). Another distinction in our study population is the high number of individuals (25.4%) with congenital heart disease. To our knowledge, the MECKI score has not, until now, been validated in this patient population. Interestingly, all but two patients diagnosed with congenital heart disease clustered in the low risk (MECKI < 10%) category, although the clinical implications of this finding require further study. The features of our patient cohort infer uniformity of the MECKI score's prognostic capabilities, irrespective of patient characteristics.

This study is also valuable in comparing the prognostic value of the MECKI score (which includes not only CPX parameters but additional clinical variables tied to renal, hematologic, and cardiac function) to a risk assessment that is based only on CPX parameters. These results support further that the MECKI score is both more sensitive and specific in predicting 2‐year HF events for patients with HF than standard CPX‐based risk assessments. By using the MECKI score, clinicians may be more able to identify patients at risk for poor outcomes and thus intervene more quickly in escalation of their care.

Limitations to this study include those inherent to a single center retrospective analysis, where confounding unmeasured factors cannot be excluded. While we observed several statistically significant findings, the study cohort was nonetheless relatively small, which limited further subgroup analyses. Additionally, we calculated VE:VCO_2_ slope to peak exercise for incorporation into the MECKI score and CPX Risk Score. Given the expected inflection in the VE:VCO_2_ curve at the respiratory compensation point, it has been proposed that measuring VE:VCO_2_ slope only to this point may provide more reliable data of improved prognostic performance (Nayor et al., 2020). However, our CPX laboratory began incorporating this new means of calculating VE:VCO_2_ slope into regular practice midway through the examined cohort (starting in 2023), so for the current analysis we used VE:VCO_2_ slope as measured to peak exercise for internal consistency and similarity to the original MECKI score publications. Similarly, for the estimation of glomerular filtration rate we used the Modification of Diet in Renal Disease equation, consistent with the original MECKI score publications. Newer equations for estimating glomerular filtration rate are now preferred (Inker et al., 2021), and the impact of adopting such equations on the performance of the MECKI score requires future investigation.

## 5 CONCLUSION

In conclusion, the MECKI score is a tool which may aid clinicians in stratifying patients undergoing CPX. In this U.S. cohort with mixed baseline characteristics, the MECKI score outperformed the summed CPX Risk score for predicting adverse HF events within 2 years. Further work is needed to clarify the potential role of the MECKI score in clinical practice.

## AUTHOR CONTRIBUTIONS

Conceived and designed research (JMC and CRT), performed experiments (AM, KD, TZ, BP, SK, JMC, and CRT), analyzed data (AM, KD, MGK, TZ, and CRT), interpreted results of experiments (AM, KD, MGK, TZ, HP, RA, JMC, and CRT), prepared figures (CRT), drafted manuscript (AM, MGK, and CRT), edited and revised manuscript (AM, KD, MGK, TZ, BP, SK, HP, RA, JMC, and CRT), approved final version of manuscript (AM, KD, MGK, TZ, BP, SK, HP, RA, JMC, and CRT).

## FUNDING INFORMATION

JMC is supported by award number K23HL159270, and CRT is supported by award number K23HL166956, both from the National Heart, Lung, and Blood Institute of the National Institutes of Health. This project was in part supported by CTSA award No. UM1TR004360 from the National Center for Advancing Translational Sciences. SK was supported by award number 23IAUST1027504 from the American Heart Association.

## CONFLICT OF INTEREST STATEMENT

No relevant conflicts of interest.

## ETHICS STATEMENT

The study was approved by the Virginia Commonwealth University institutional review board, with a waiver of consent due to the retrospective nature of the study. The study was performed in accordance with the Declaration of Helsinki.

## DISCLAIMERS

The contents of this manuscript are solely the responsibility of the authors and do not necessarily represent the official views of the funding agencies.

## Supporting information


Appendix S1.

## Data Availability

Source data for this study are not publicly available due to privacy or ethical restrictions. The source data are available to verified researchers upon request by contacting the corresponding author.
