# Supplementary material for: Prognostic utility of the MECKI score in a mixed United States cohort
Source: Physiol Rep. 2026 Feb 11;14(3):e70770. doi: 10.14814/phy2.70770 (PMC12894076; doi:10.14814/phy2.70770)
Supplement: Supplementary file 1 — Appendix S1. [file PHY2-14-e70770-s001.docx]

**Supplemental Figure S1 – Kaplan Meier Event-Free Survival Curves Using Ideal Cutoff Values in the Total Cohort**

**
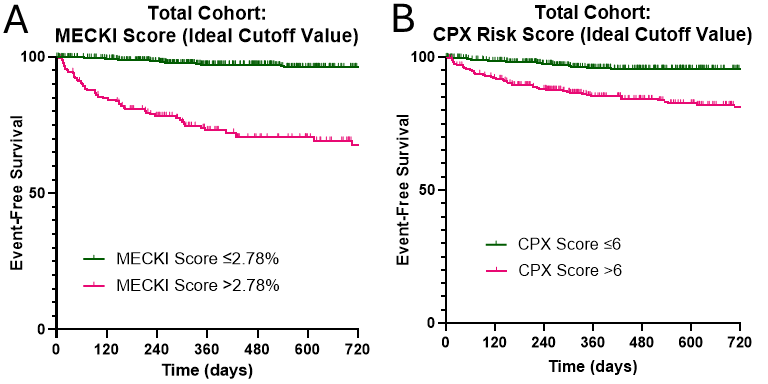
**

Both the MECKI score (A) and CPX Risk score (B) allowed for discrimination of risk among the entire cohort using ideal cutoff values according to Youden’s index (2.78% and 6, respectively).

**Supplemental Figure S2 – Kaplan Meier Event-Free Survival Curves Using Ideal Cutoff Values in the Subset of Patients with HFrEF**

**
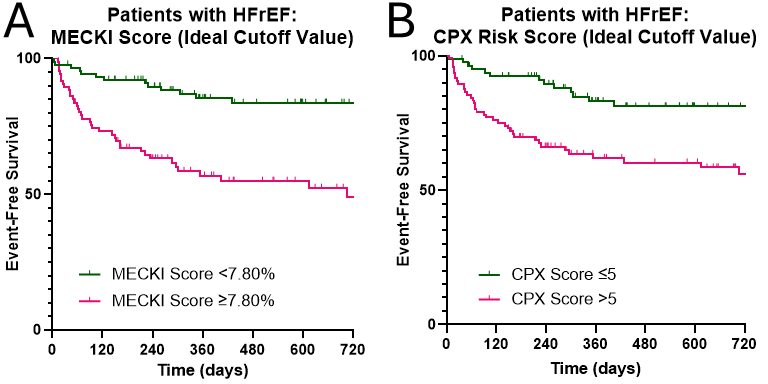
**

The MECKI score (A) and CPX Risk score (B) discriminated risk of adverse heart failure events among patients with heart failure with reduced ejection fraction using ideal cutoff values according to Youden’s index (7.80% and 5, respectively).

**Supplemental Table S1 – MECKI and CPX Risk Score Calculations**

|  | Formulas |
| --- | --- |
| Percent predicted peak VO_2_ | Males: (100 * VO_2_ peak) / ([Height – Age] * 20)  Females: (100 * VO_2_ peak) / ([Height – Age] * 14) |
| Estimated glomerular filtration rate | Males: 186.3 * (serum creatinine)^−1.154^ * (Age)^−0.203^  Females: 186.3 * (serum creatinine)^−1.154^ * (Age)^−0.203^ * 0.75 |
| MECKI score | 100 * *e^c^*/(1 + *e^c^*) where *c* = 10.3464 + (−0.0262 * ppVO_2_) + (0.0472 * VE/VCO_2_ slope) + (−0.1086 * hemoglobin) + (−0.0615 * serum sodium) + (−0.0699 * LVEF) + (−0.0136 * eGFR) |
| CPX risk score | +7, if VE/VCO_2_ slope $\geq$ 34  +5, if heart rate recovery $\leq$ 6 beats  +3, if oxygen uptake efficiency slope $\leq$ 1.4  +3, if rest partial pressure end tidal CO_2_ < 33 mmHg  +2, if VO_2_ peak $\leq$14.4 mL •kg^-1^•min^-1^ |

**Supplemental Table S2 – Performances of the MECKI and CPX Risk Scores Using Optimal Cutoff Values in the Entire Cohort**

|  |  | **MECKI Score** | | **CPX Score** | |
| --- | --- | --- | --- | --- | --- |
|  |  | >2.78 | ≤2.78 | >6 | ≤6 |
| **Event at 2 Years** | **Yes** | 60 | 18 | 59 | 19 |
|  | **No** | 132 | 593 | 284 | 441 |
|  |  | Sensitivity: 77% | | Sensitivity: 76% | |
|  |  | Specificity: 82% | | Specificity: 61% | |
|  |  | **Accuracy: 81%** | | **Accuracy: 62%** | |

**Supplemental Table S3 – Performances of the MECKI and CPX Risk Scores Using Optimal Cutoff Values in the Subset of Patients with HFrEF**

|  |  | **MECKI Score** | | **CPX Score** | |
| --- | --- | --- | --- | --- | --- |
|  |  | ≥7.80 | <7.80 | >5 | ≤5 |
| **Event at 2 Years** | **Yes** | 44 | 14 | 44 | 14 |
|  | **No** | 46 | 84 | 58 | 72 |
|  |  | Sensitivity: 76% | | Sensitivity: 76% | |
|  |  | Specificity: 65% | | Specificity: 55% | |
|  |  | **Accuracy: 68%** | | **Accuracy: 62%** | |
